# Transcriptional profiles of vaccine-induced protection in bovine herpesvirus-1 and *Mycoplasma bovis*-challenged bison

**DOI:** 10.3389/fvets.2025.1667623

**Published:** 2025-09-16

**Authors:** Anna K. Goldkamp, Bryan S. Kaplan, Harish Menghwar, Carly R. Kanipe, Paola M. Boggiatto, Lauren S. Crawford, Steven C. Olsen, Robert E. Briggs, Fred M. Tatum, Rohana P. Dassanayake, Eduardo Casas

**Affiliations:** ^1^Ruminant Diseases and Immunology Research Unit, National Animal Disease Center, United States Department of Agriculture, Agricultural Research Service, Ames, IA, United States; ^2^ARS Research Participation Program, Oak Ridge Institute for Science and Education (ORISE), Oak Ridge, TN, United States; ^3^Infectious Bacterial Diseases Research Unit, National Animal Disease Center, Agricultural Research Service, United States Department of Agriculture, Ames, IA, United States

**Keywords:** animal health, wild species, infectious disease, transcriptome, *Mycoplasma bovis*

## Abstract

**Introduction:**

*Mycoplasma bovis* causes chronic respiratory disease with high mortality rates in American bison (*Bison bison*). A recent study showed that a subunit vaccine containing *M. bovis* elongation factor thermal unstable (EFTu) and heat shock protein 70 (Hsp70) antigens induced immunity and enhanced protection in bison, resulting in reduced lung lesions and bacterial loads following experimental *M. bovis* challenge. This study aimed to characterize the transcriptional responses underlying this protection in vaccinated (*n* = 5) compared to unvaccinated control (*n* = 4) bison following *M. bovis* infection.

**Methods:**

Two doses of vaccines were administered on day 0 and at 21 days post-vaccination (DPV), followed by intranasal inoculation with bovine herpesvirus-1 (BHV-1) at 36 DPV and *M. bovis* at 40 DPV. RNA sequencing was performed on liver, palatine tonsil (PT), retropharyngeal lymph node (RPLN), tracheobronchial lymph node (TBLN), spleen, and whole blood samples. Blood was collected at 1st vaccination (Day 0), 2nd vaccination (21 days post-vaccination), BHV-1 inoculation (36 DPV), *M. bovis* inoculation (40 DPV), and 1 week post *M. bovis* inoculation (47 DPV).

**Results and discussion:**

The greatest number of differentially expressed transcripts (DETs) (≤0.05 FDR) were found in blood at 36 DPV (123 total DETs) and in spleen (57 DETs). At 36 DPV, vaccinated animals showed upregulation of transcripts involved in in cell adhesion, T-helper cell (Th1/Th2/Th17) differentiation, and antigen processing and presentation. This signifies a robust response to the 2nd vaccine dose, which caused increased expression of *CD3E*, *CD4*, and *CD8B* correlating to increased T cell proliferation. Notably, transcription factors *TBX21* and *GATA3* were upregulated in vaccinated animals. Spleen-specific regulation included transcripts involved in innate immune response, such as *LGALS3* and *GBP-1*. These findings highlight the robust immune response induced by the vaccine, particularly through T-cell mediated responses, demonstrating its potential to enhance protective immunity against *M. bovis* in bison.

## Introduction

*Mycoplasma bovis* is a bacterium without a cell wall (or mollicutes) frequently associated with the bovine respiratory disease complex (BRDC) in cattle (1). In cattle, *M. bovis* is considered a secondary pathogen appearing after or alongside primary infections (2). Stressors, such as weaning, transport, or a concurrent viral infection, can lead to a compromised immune system that allows *M. bovis* to colonize the lower respiratory tract and cause BRDC (3). *M. bovis* is also known to cause mastitis in dairy cattle (4). Although *M. bovis* has long been recognized as a prominent pathogen in cattle, it has recently emerged as a significant threat to North American bison populations. In bison, *M. bovis* can act as a primary pathogen causing high mortality epizootics of casonecrotic pneumonia frequently associated with pharyngitis, laryngitis, polyarthritis, and other pathologies (5–7).

Transcriptome studies have been used to study immune responses in the context of animal health. Alterations in gene expression have been observed in cattle infected with pathogens of the BRDC, such as *M. bovis*, *Mannheimia haemolytica*, bovine herpesvirus-1 (BHV-1), and bovine viral diarrhea virus (8–10). Pathogens associated with mastitis infection (*Staphylococcus aureus*) and blood-borne infection (bovine leukemia virus) also modulate host gene expression profiles, reflecting immune activation and disease pathogenesis (11, 12). Biomarkers of infection and inflammation can be used for detection and outcome prediction of BRDC. However, transcriptional alterations in bison in response to infection or vaccination have not been characterized.

We previously reported an injectable subunit vaccine containing recombinant elongation factor thermal unstable (EFTu) and heat shock protein 70 (Hsp70) can confer protection against *M. bovis* infection in bison (13). Vaccination resulted in reductions in lung pathology, lung bacterial loads, and detection of bacteria in the joints. Bison that received vaccine had a robust antibody response in addition to a T cell response characterized by the development of antigen specific CD8^+^ and ϒδ^+^ T cells. Additionally, we observed increased blood neutrophil counts in half of the unvaccinated control bison following challenge (13). To deepen our understanding of *M. bovis* pathogenic mechanisms and the host immune response to vaccination, the associated transcriptional responses were investigated. We hypothesize that vaccination with recombinant *M. bovis* antigens enhances protective immunity in bison by priming transcriptional pathways involved in T-cell activation. The objective of the current study was to assess the impacts of vaccination on gene expression profiles across various tissues (liver, palatine tonsil, retropharyngeal lymph node, tracheobronchial lymph node, and spleen) and sequentially collected blood samples in bison.

## Materials and methods

### Animal welfare

Animals were housed and samples collected in accordance with the Animal Welfare Act Amendments (7 U. S. Code e § 2,131 to § 2,156). All protocols and procedures were approved by the Institutional Animal Care and Use Committee of the National Animal Disease Center, Ames, IA (ARS-22-1035) before the onset of the study. Animal caretakers, veterinarians, and scientific staff performed animal monitoring and record keeping. Animals were humanely euthanized by intravenous sodium pentobarbital following the per-label dose and the discretion of the clinical veterinarian.

### Animal origin

The animals were purchased from a commercial facility by USDA. The present study uses tissue and blood samples collected from the same animals in a previous study that has been published (13).

### Animal study

Nine bison (~1–5 years old) were randomly assigned to one of two treatment groups: unvaccinated control (*n* = 4) or vaccinated (*n* = 5). An injectable subunit vaccine was formulated by mixing 100 μg of recombinant EFTu and 100 μg of Hsp70 with an oil-in-water emulsified adjuvant (Emulsigen®-D) as previously described (13, 14). Unvaccinated control animals were given a mock vaccine containing only emulsified adjuvant. Two doses of either vaccine or mock vaccine (2 mL per dose) were given at day 0, and at 21 days post-vaccination (DPV). Animals were inoculated with bovine herpesvirus-1 (BHV-1) at 36 DPV and with *M. bovis* at 40 DPV using an intranasal mucosal atomization device. Inoculums were prepared as previously described (5). Animals were euthanized between 51–65 DPV. Euthanasia and event summary tables can be found in Supplementary Table S1. Peripheral blood was collected by venipuncture and frozen in PAXGene tubes at 5 time points: Day 0 (1st vaccination), 21 DPV (2nd vaccination), 36 DPV (BHV-1 inoculation), 40 DPV (*M. bovis* inoculation), and 47 DPV (1 week post *M. bovis* inoculation). Blood samples were taken at the same time for all animals and collected directly before vaccination or inoculation events. Liver, palatine tonsil (PT), retropharyngeal lymph node (RPLN), tracheobronchial lymph node (TBLN), and spleen were collected at necropsy and immediately stored in RNAlater (Thermo Fisher Scientific, Waltham, MA, United States).

### RNA isolation

Total RNA was extracted and purified using the MagMAX™ mirVana™ Total RNA Isolation Kit (Thermo Fisher Scientific) according to manufacturer’s instructions. Quality and concentration of total RNA samples was evaluated using the Series II RNA 6000 Nano LabChip® kit (Agilent Technologies, Santa Clara, CA, United States) and samples with an RNA integrity number of ≥7.5 were used for subsequent library preparation.

### Library preparation and sequencing

The NEBNext Poly(A) mRNA Magnetic Isolation Module was used for mRNA selection from extracted total RNA and libraries were prepared using the NEBNext Ultra II RNA library prep kit (New England Biolabs, Ipswitch, MA, United States) according to manufacturer’s instructions. Library index incorporation was done using the NEBNext Multiplex Oligos for Illumina kit (New England Biolabs). Libraries were pooled in equal concentration and stored at −20°C until sequencing using the NovaSeq6000 system on S2 flow cells with 150-cycle paired end reads (2 × 150 bp) at the Iowa State University DNA sequencing facility.

### RNAseq data processing

Quality of raw reads was assessed with FastQC (version 0.12.1). Adapter sequences were removed and low-quality bases were trimmed (quality-cutoff of 30 and minimum-length ≥60) using Cutadapt (version 4.0). The Bison genome fasta file and annotation files (Bison_UMD1.0) were downloaded from Ensembl. For read alignment and quantification of transcript abundance, the STAR-RSEM pipeline was used (STAR version 2.7.10b; RSEM version 1.3.3). Mapping indices were created and trimmed reads were mapped with STAR. Transcript expression was quantified with rsem-calculate-expression and rsem-generate-data-matrix was used to generate the transcript count matrix.

### Differential expression analysis

The software DESeq2 (version 1.44.0) was used for differential expression analysis. The DESeqDataSetFromMatrix and estimateSizeFactors functions were used to create a DESeq2 object and normalize counts with the median ratio method, respectively. Transcripts with at least 5 normalized counts in at least 2 samples were kept for further analysis. The Wald test was used to generate baseMean, log2FoldChange, log fold change standard error, and adjusted *p*-values. The Benjamini-Hochberg procedure was used by DESeq2 for *p*-value adjustment in order to control the false discovery rate. Transcripts were deemed significant with an adjusted *p*-value < 0.05.

Correlation heatmaps for all samples were generated with pheatmap (version 1.0.12). Volcano plots and heatmaps were generated using the R packages EnhancedVolcano (version 1.22.0) and ggplot2 (version 3.5.1). Venn diagrams were generated using ggVennDiagram (version 1.5.2). Gene ontology and KEGG enrichment analysis of DETs was performed with DAVID.

### Weighted gene co-expression network analysis (WGCNA)

The WGCNA R package (version 1.73) was used to identify modules of highly correlated transcripts connected to vaccination status (15). The top (75% quantile) transcripts for expression variance were identified using the quantile function and used for module identification. The weighted network adjacency matrix was calculated from the expression data based on Pearson correlation coefficients using the adjacency function and a soft-thresholding power of 5 was used to achieve a scale free topology index of 0.85. The adjacency matrix was converted into a topological overlap matrix with the TOMsimilarity function and hierarchical clustering grouped transcripts into modules with a minimum size of 10 transcripts. Eigen values were correlated to vaccination status using cor and corPvalueStudent functions of WGCNA. Network files were exported and visualized using Cytoscape (version 3.10.3).

## Results

### Overview of RNAseq data

To quantify transcript expression, raw reads were processed and aligned to the bison reference genome (Bison_UMD1.0). An average of ~36 million raw reads were obtained per sample. Adapter and quality trimming yielded an average of ~27 million trimmed reads per sample. In tissues samples, alignment rates ranged from 84.6% (PT) to 88.82% (RPLN). In blood, alignment rates ranged from 82.7% (2nd vaccination) to 87.5% (1st vaccination).

**Figure 1:**
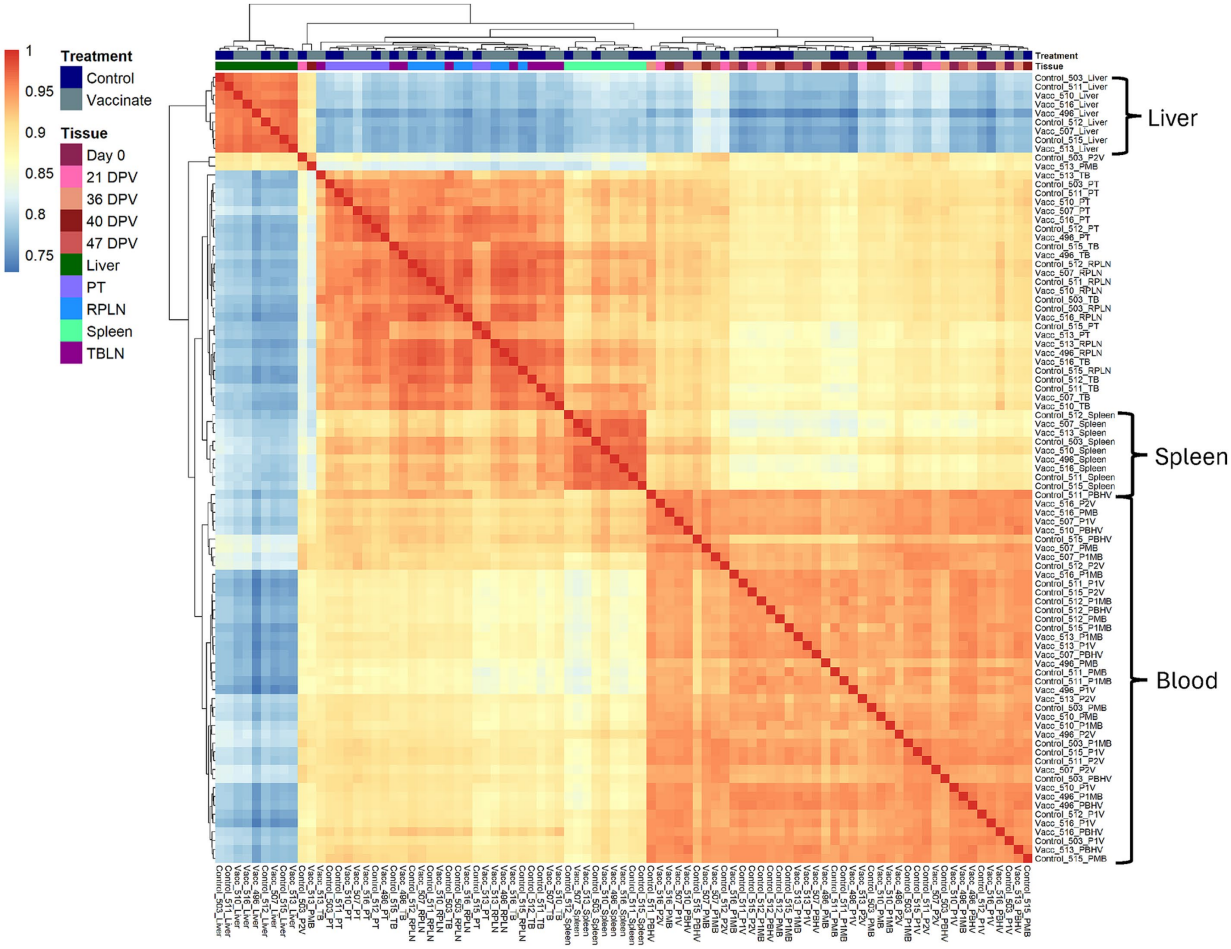
Dendrogram correlation heatmap across liver, PT, RPLN, TBLN, spleen, and blood (0 DPV, 21 DPV, 36 DPV, 40 DPV, 47 DPV) showing clustering of samples based on gene expression. Correlation scale is indicated by red (highest correlation) and blue (lowest correlation) colors. Treatment colors are shown in blue (Control) or gray (Vaccinate). Sample type is shown in different colors. The samples were collected from unvaccinated control (*n* = 4) and vaccinated (*n* = 5) animals.

Liver, spleen, and blood samples separated into distinct clusters with high correlations across samples (*R* > 0.9), whereas PT, RPLN, and TBLN often clustered together indicating greater similarity in gene expression (Figure 1).

### Number of differentially expressed transcripts

**Figure 2:**
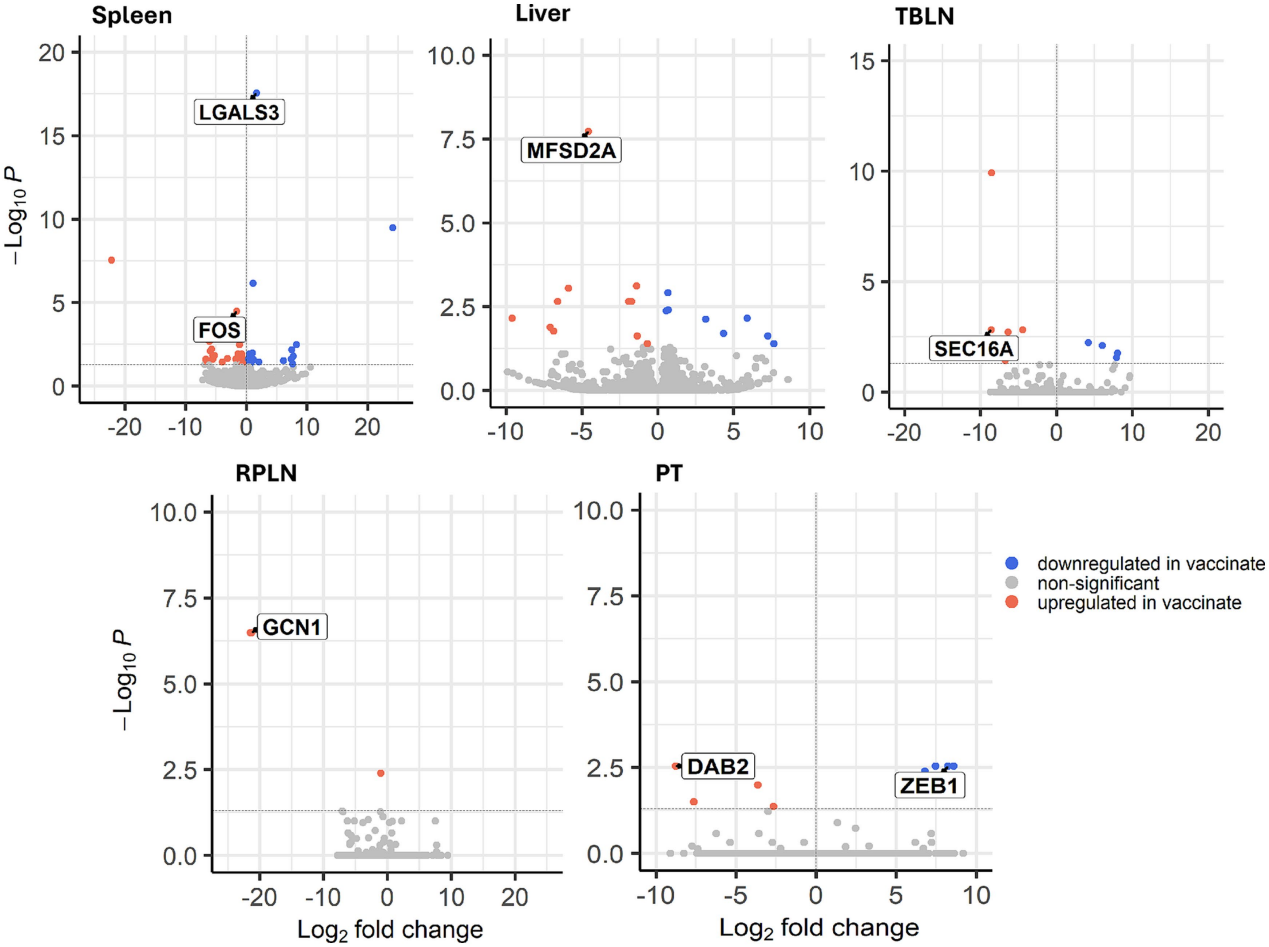
Volcano plot of differentially expressed transcripts (DETs) between control and vaccinate in liver, tracheobronchial lymph node (TBLN), retropharyngeal lymph node (RPLN), palatine tonsil (PT), and spleen. Log2 fold change is shown on the *x*-axis and -log10 adjusted *p*-value on the *y* axis. Transcripts downregulated in vaccinate are shown in blue and transcripts upregulated in vaccinate are shown in pink. Non-significant transcripts are shown in grey. LGALS3 = Galactin-3; FOS = Fos Proto-Oncogene, AP-1 Transcription Factor; MFSD2A = major facilitator super family domain containing 2a; SEC16A = SEC16 homolog A; GCN1 = GCN activator of EIF2AK4; DAB2 = Disabled-2; ZEB1 = Zinc finger E-box binding homeobox 1. The samples were collected from unvaccinated control (*n* = 4) and vaccinated (*n* = 5) animals.

A summary of differentially expressed transcripts between control and vaccinated animals in all tissues and time points is shown in Table 1. The greatest number of differentially expressed transcripts (DETs) within tissues was found in spleen, where 57 DETs were identified. Galactin-3 (*LGALS3*) was downregulated and Fos Proto-Oncogene, AP-1 Transcription Factor (*FOS*) was upregulated in spleen of vaccinated animals (Figure 2). The 2nd highest number of DETs in tissue was found in liver, where major facilitator super family domain containing 2a (*MFSD2A*) was upregulated in vaccinated animals compared to control (16). The number of DETs in other tissues ranged from 3 (RPLN) to 12 (TBLN). DETs included SEC16 homolog A (*SEC16A*), which was upregulated in TBLN of vaccinated animals (17). Upregulation of general control non-derepressible protein 1 (*GCN1*) was observed in RPLN of vaccinated animals. A regulator of interferon signaling, zinc finger E-box binding homeobox 1 (*ZEB1*), was downregulated and an immunoregulatory molecule, Disabled-2 (*DAB2*), was upregulated in PT of vaccinated animals (18–20).

**Table 1:** Number of up- and down-regulated differentially expressed transcripts between Control and Vaccinate animals.

**Tissue**	**Number of differentially expressed transcripts**		
	**Up-regulated**	**Down-regulated**	**Total**
Liver	9	17	26
TBLN	4	8	12
RPLN	0	3	3
PT	4	4	8
Spleen	26	31	57
Blood			
0 DPV	3	8	11
21 DPV	5	7	12
36 DPV	40	83	123
40 DPV	12	21	33
47 DPV	10	3	13

Up-regulated and down-regulated columns indicate the number of differentially expressed transcripts in control (*n* = 4) compared to vaccinate (*n* = 5). An adjusted *p*-value of ≤0.05 was used as a threshold for significance.

**Figure 3:**
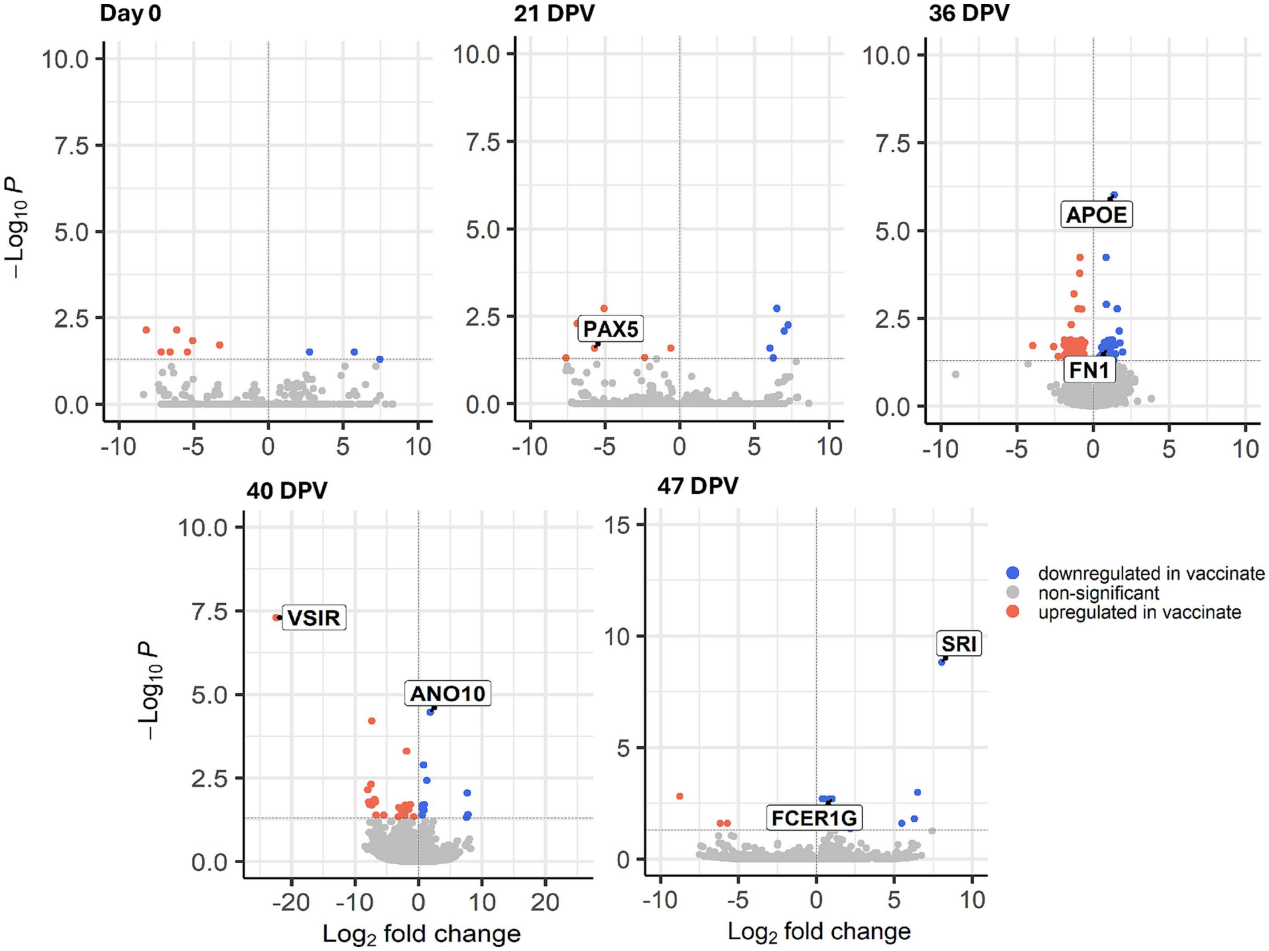
Volcano plot of differentially expressed transcripts (DETs) between control and vaccinate in 0 days post-vaccination (DPV), 21 DPV, 36 DPV, 40 DPV, 47 DPV. Log2 fold change is shown on the *x*-axis and –log10 adjusted *p*-value is on the *y* axis. Transcripts downregulated in vaccinate are shown in blue and transcripts upregulated in vaccinate are shown in pink. Non-significant transcripts are shown in gray. PAX5 = Paired box 5; APOE = Apolipoprotein E; FN1 = Fibronectin 1; VSIR = V-set immunoregulatory receptor; ANO10 = Anoctamin 10; SRI = Sorcin; FCER1G = Fc fragment of IgE receptor Ig. The samples were collected from unvaccinated control (*n* = 4) and vaccinated (*n* = 5) animals.

The number of DETs between control vs. vaccinated animals in blood ranged from 11 total DETs (Day 0) to 123 total DETs (36 DPV) (Table 1). The transcription factor paired box 5 (*PAX5*) facilitates differentiation of B cells and was upregulated in vaccinated animals at 21 DPV as shown in Figure 3 (21). Transcripts related to heparin binding, such as apolipoprotein E (*APOE*) and fibronectin 1 (*FN1*) were downregulated in vaccinated animals at 36 DPV (22, 23). At 40 DPV, v-set immunoregulatory receptor (*VSIR*) was upregulated in vaccinated animals (24). Anoctamin 10 (*ANO10*) was downregulated in vaccinated animals compared to control (25). Sorcin (*SRI*) and Fc fragment of IgE receptor Ig (*FCER1G*) were downregulated at 1 week post *M. bovis* infection (47 DPV) in vaccinated animals (26, 27). DESeq2 results for all samples can be found in Supplementary Tables S2, S3.

### Shared differentially expressed transcripts (DETs) across tissues and blood time points

**Figure 4:**
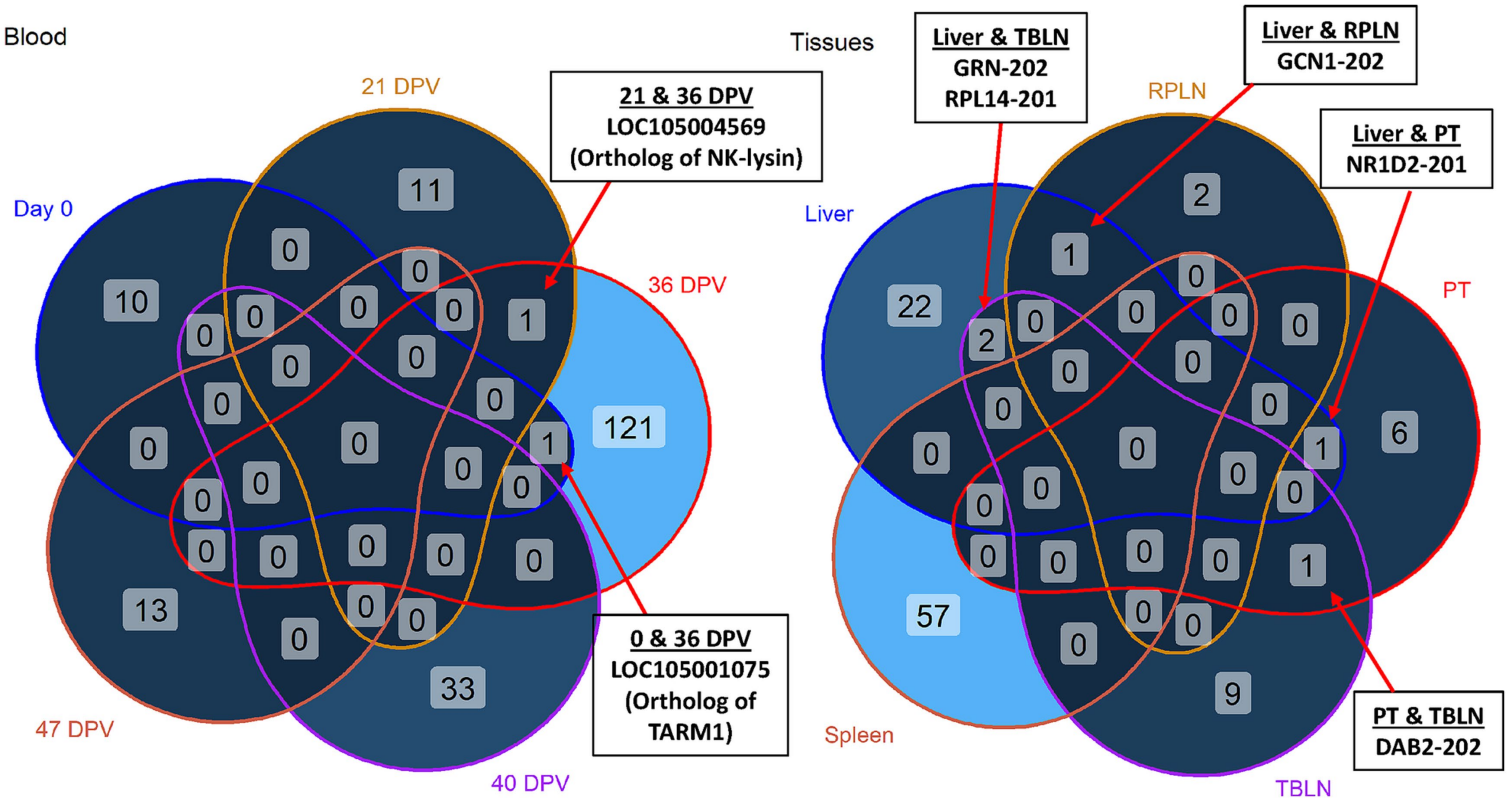
Venn diagrams of differentially expressed transcripts (DETs). Shared and unique DETs across day 0, 21 days post-vaccination (DPV), 36 DPV, 40 DPV, 47 DPV. Shared and unique DETs across liver, retropharyngeal lymph node (RPLN), palatine tonsil (PT), tracheobronchial lymph node (TBLN), and spleen. Shared DETs are indicated by arrows. TARM1 = T cell-interacting, activating receptor on myeloid cells 1; GRN = granulin precursor; RPL14 = ribosomal protein L14; GCN = GCN1 activator of EIF2AK4; NR1D2 = nuclear receptor subfamily 1 group D member 2; DAB2 = DAB adapter protein 2. The samples were collected from unvaccinated control (*n* = 4) and vaccinated (*n* = 5) animals.

Out of 190 DETs, 178 were unique to day of vaccination in blood. However, LOC105004569 (ortholog of NK-lysin) and LOC105001075 (ortholog of T cell-interacting activating receptor on myeloid cells 1/*TARM1*) were shared across more than one time point (Figure 4, left). The antimicrobial peptide, NK-lysin, is produced by T lymphocytes and natural killer cells, and was upregulated in vaccinated animals at 21 and 36 DPV (28). The leukocyte receptor, *TARM1*, is critical for macrophage activation and was upregulated in vaccinated animals at day 0 and 36 DPV (29).

In tissue comparisons, DETs in liver overlapped with DETs in TBLN, RPLN, and PT (Figure 4, right). Granulin precursor (*GRN*) and ribosomal protein L14 (*RPL14*) were upregulated in liver and TBLN of vaccinated animals. The gene *GCN1* was downregulated in liver and upregulated in RPLN of vaccinated animals. Nuclear receptor subfamily 1 group D member 2 (*NR1D2*) was downregulated in liver and PT of vaccinated animals (30). A negative regulator of Toll-like receptor signaling, *DAB2*, was upregulated in PT and downregulated in TBLN of vaccinated animals (31).

### Gene ontology and KEGG enrichment analysis

As shown in Table 2, significant enrichment of DETs was observed in spleen and liver. No significant enrichment was observed in PT, TBLN, or RPLN. DETs in spleen, such as T-complex 1 (*TCP1*) and prostaglandin E synthase 3 (*PTGES3*), were downregulated in vaccinated animals and enriched in biological processes of protein stabilization and positive regulation of telomere maintenance. There was a tendency for enriched innate immune response (*p*-value = 0.06), where *LGALS3* and *LOC105003308* (Ortholog of interferon-induced guanylate-binding protein/*GBP-1*) were downregulated in vaccinate spleen and ADAM metallopeptidase domain 15 (*ADAM15*) was upregulated. In liver, protein tyrosine phosphatase non-receptor type 6 (*PTPN6*) and beta-transducin repeat containing E3 ubiquitin protein ligase (*BTRC*) were upregulated in vaccinated animals and enriched in the negative regulation of T cell receptor signaling pathway.

**Table 2:** The significant pathways, biological processes, and molecular functions enriched with differentially expressed transcripts (DETs) in spleen and liver.

**Tissue**	**Category**	**Term**	***p*-value**	**Transcripts**
Spleen	Biological Process	Protein stabilization	1.38E-02	*TCP1-201, PPAP2B-201, PTGES3-201*
	Biological Process	Positive regulation of telomere maintenance via telomerase	2.36E-02	*TCP1-201, PTGES3-201*
	Molecular Function	Lipid transporter activity	3.89E-02	*ABCA9-201, NPC1-202*
Liver	Biological Process	Negative regulation of T cell receptor signaling pathway	6.50E-03	*PTPN6-202, BTRC-202*

**Figure 5:**
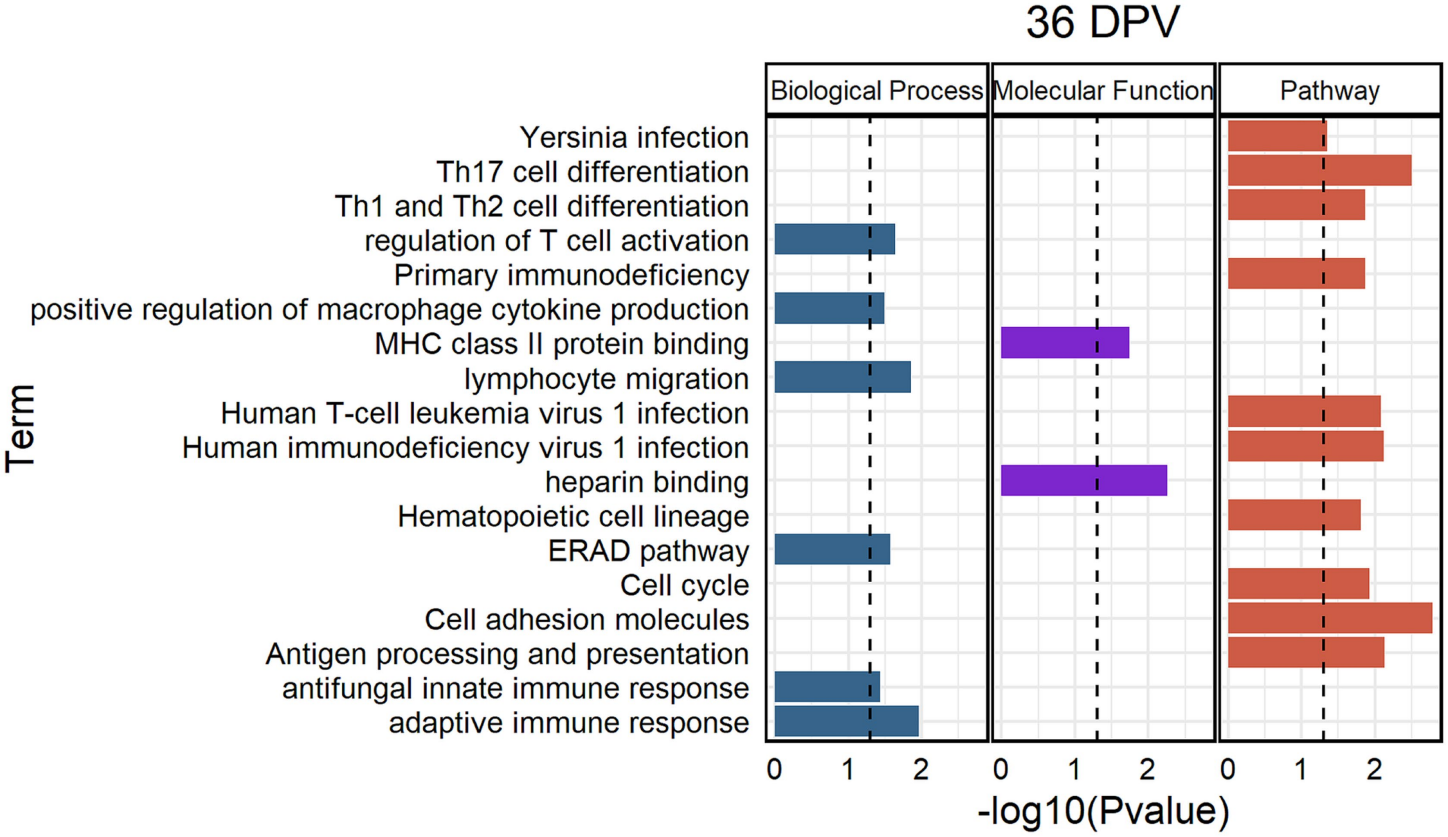
Significantly enriched biological processes, molecular functions, and pathways at 36 days post vaccination (DPV). Categories are depicted in different colors. The *x*-axis shows log10 *p*-value and the *y*-axis shows the enriched term. The dashed line indicates a *p*-value threshold of 0.05. The samples were collected from unvaccinated control (*n* = 4) and vaccinated (*n* = 5) animals.

**Figure 6:**
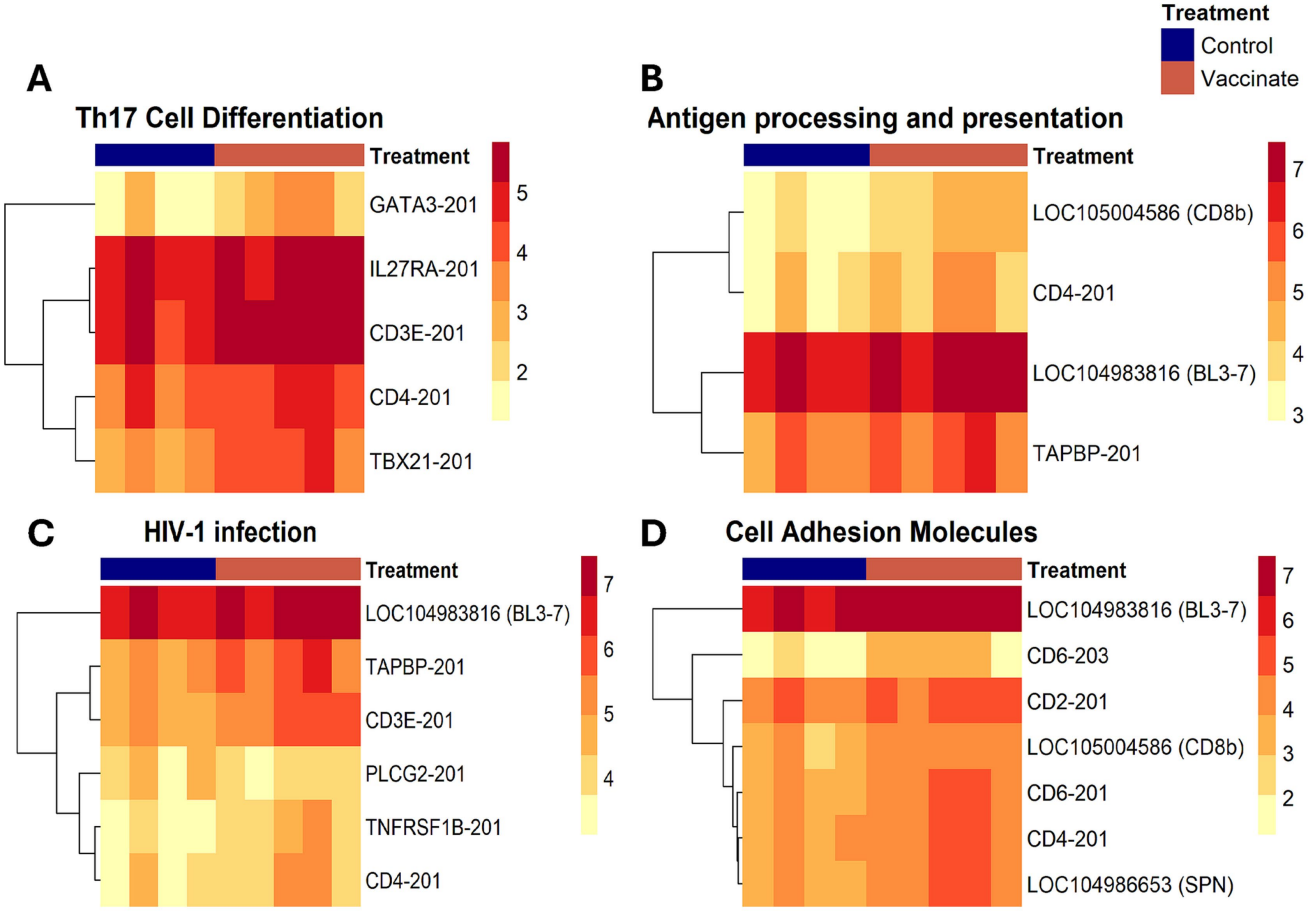
Heatmap visualization of top significantly enriched pathways at 36 days post-vaccination. Differentially expressed transcripts (DETs) were significantly enriched in **(A)** Th17 cell differentiation, **(B)** antigen processing and presentation, **(C)** HIV-1 infection, and **(D)** cell adhesion molecules. The color scale shows the log counts per million (logCPM) expression level of the transcripts within each sample. The samples were collected from unvaccinated control (*n* = 4) and vaccinated (*n* = 5) animals.

The greatest number of significantly enriched gene ontology terms and pathways was found at 36 DPV (Figure 5). The most significantly enriched pathways were cell adhesion molecules, Th17 cell differentiation, antigen processing and presentation, and HIV-1 infection pathways. Infiltrated lymphocyte markers (GATA binding protein 3/*GATA3*, Interleukin 27 receptor subunit alpha/*IL27RA*, *CD3E*, *CD4,* and T-box transcription factor 21/*TBX21*) were enriched in Th17 cell differentiation and upregulated in vaccinated animals at 36 DPV (Figure 6A). The ortholog of bovine leukocyte antigens class I major histocompatibility complex (MHC), alpha chain BL3-7 (LOC104983816) and a binding partner of MHC Class I molecules, TAP binding protein (*TAPBP*), were upregulated in vaccinated animals and enriched in antigen processing and presentation (Figure 6B). Phospholipase C, gamma-2 (*PLCG2*) was downregulated and tumor necrosis factor receptor superfamily member 1B (*TNFRSF1B*) was upregulated in vaccinated animals and enriched in HIV-1 infection (Figure 6C). Upregulation of cell adhesion DETs was found, such as *CD2*, *CD6*, LOC105004586 (Ortholog of *CD8b*), and LOC104986653 (Ortholog of Sialophorin/*SPN*) at 36 DPV (Figure 6D).

At 40 DPV (or 4 days post-BHV-1 challenge), VPS50 subunit of EARP (*VPS50*) and SEC31 Homolog A (*SEC31A*) were upregulated in vaccinated animals and enriched in protein transport. In addition, two upregulated (Fibulin 5/*FBLN5* and SPARC Like 1/*SPARCL1*) and two downregulated (RAS Guanyl Releasing Protein 4/*RASGRP4* and S100 Calcium Binding Protein A4/*S100A4*) DETs in vaccinated animals were enriched in calcium ion binding at 40 DPV.

### Transcript co-expression at 40 and 47 DPV

**Figure 7:**
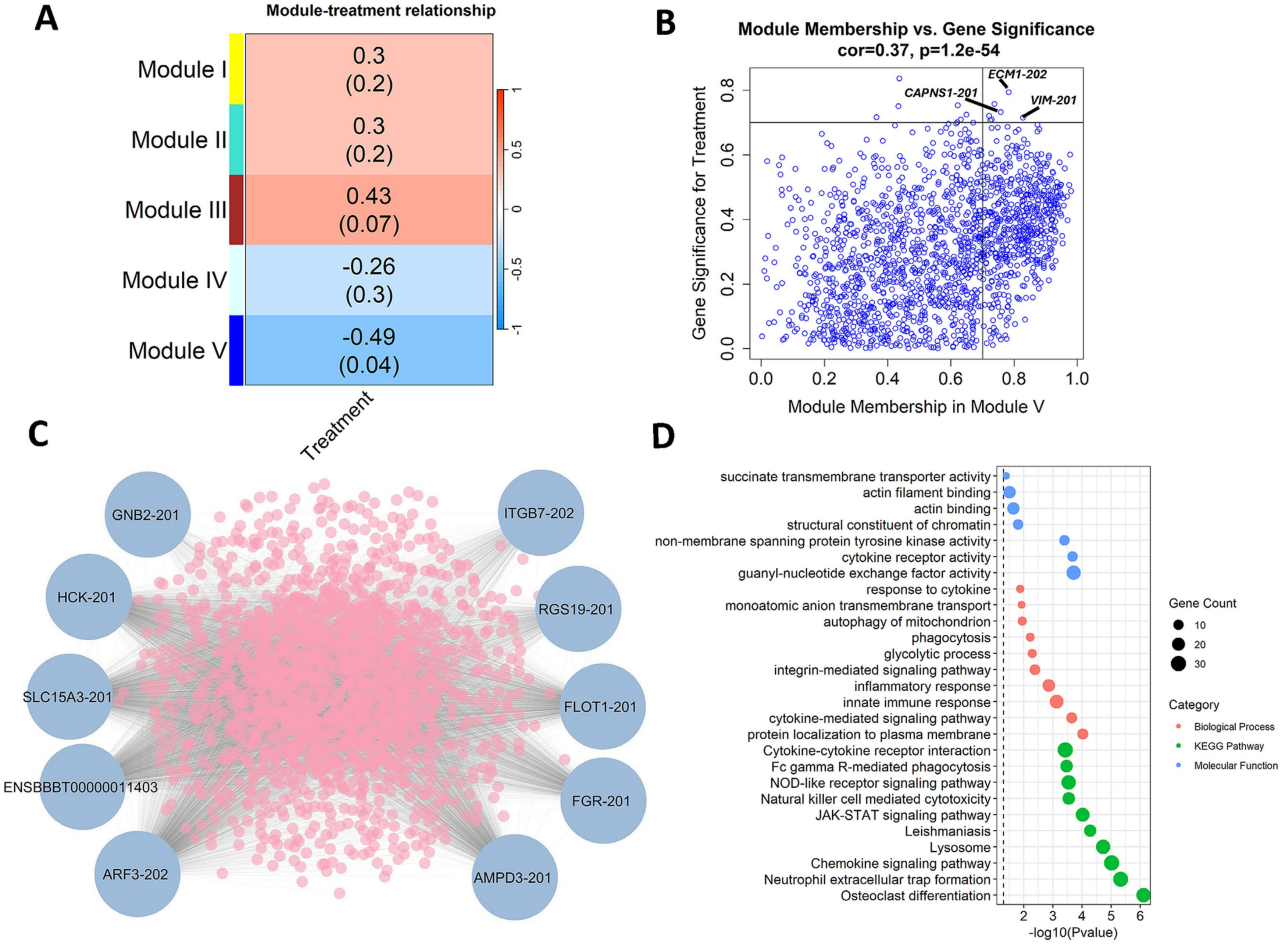
Weighted gene co-expression analysis (WGCNA) reveals co-expression at 40 days post vaccination (DPV) and 47 DPV. **(A)** Module treatment correlation matrix displaying identified modules of co-expressed transcripts. **(B)** Correlation between module membership and gene significance for Module V transcripts. Transcripts with a module membership and significance greater than 0.7 are highlighted. **(C)** Top 10 transcripts with high connectivity to the expression of other transcripts in Module V. **(D)** Enrichment of Module V co-expressed transcripts in biological processes, KEGG pathways, and molecular functions. The samples were collected from unvaccinated control (*n* = 4) and vaccinated (*n* = 5) animals.

Weighted co-expression analysis identified 5 modules of co-expressed transcripts with correlations to treatment group at 40 and 47 DPV. Of these modules, Module V displayed a significant (*p*-value < 0.05) and intermediate, negative correlation (R = −0.49) with vaccinated animals (Figure 7A). Extracellular matrix protein 1 (*ECM1*), vimentin (*VIM*), and calpain small subunit 1 (*CAPNS1*) were among the transcripts with the highest significance (*R* > 0.7) for vaccination status and connectivity to other transcripts in the module (Figure 7B). The transcripts with the highest overall module membership in Module V included G protein subunit beta (*GNB2*), Src Family Tyrosine Kinases (*HCK* and *FGR*), solute carrier family 15 member 3 (*SLC15A3*), integrin subunit beta 7 (*ITGB7*), and flotillin 1 (*FLOT1*) as shown in Figure 7C. The most significant KEGG pathways enriched with the co-expressed transcripts in Module V included osteoclast differentiation, neutrophil extracellular trap formation, and chemokine signaling pathway (Figure 7D). The most significant enrichment for biological processes and molecular functions included protein localization to plasma membrane, cytokine-mediated signaling pathway, innate immune response, guanyl-nucleotide exchange factor activity, and cytokine receptor activity (Figure 7D).

## Discussion

*Mycoplasma bovis* is an etiologic agent of respiratory disease in American bison that is frequently associated with high morbidity and mortality (6, 32). Due to the significant economic and herd health impacts of *M. bovis* outbreaks in bison, effective intervention strategies are urgently needed. We previously reported an evaluation of the efficacy of an injectable, adjuvanted subunit vaccine containing EFTu and Hsp70 (13). In the current study, we conducted a transcriptome analysis across serially collected blood and tissues from the vaccinated and *M. bovis* challenged bison to examine host response to vaccination and better understand the molecular mechanisms mediating the partial protection observed against *M. bovis*.

T cell-mediated immunity is crucial for protection against intracellular and extracellular pathogens. While DETs at day 0 represent animal to animal variation, DETs at other time points were associated with heparin binding, immune response, and immune signaling. Vaccination resulted in an upregulation of transcripts associated with Th1, Th2, and Th17 cell differentiation with the greatest difference in gene expression occurring at approximately 2 weeks after the 2^nd^ vaccine dose (36 DPV), suggesting a multifaceted T cell response. The adjuvant utilized in the current study was Emulsigen®-D containing dimethyldioctadecyl ammonium bromide (DDA), which is effective in inducing both systemic antibody and T-cell responses (33). Our previous work demonstrated that *in vitro* stimulation of peripheral blood mononuclear cells with recombinant EFTu or Hsp70 increased proliferation of CD4, CD8, and γδ T cells in vaccinated animals compared to control with a peak at 36 DPV (13). An increase in cytotoxic and helper T cell responses was mirrored in the blood transcriptome data as *CD3E*, *CD4*, and *CD8B* were upregulated in vaccinated animals compared to controls. The upregulation of transcription factors *TBX21* and *GATA3* in vaccinated animals also has implications for enhanced protection. In Th2 cells, *GATA3* is selectively upregulated and binds to GATA motifs for Th2-specific expression to enhance humoral immunity (34). In Th1 cells, it is believed that the co-expression of T-bet (encoded by *TBX21*) sequesters *GATA3* away from Th2 genes and induces *GATA3* binding at Th1-specific sites (35). Thus, vaccination may stimulate CD4 T-cell polarization toward a Th1 phenotype by upregulating *TBX21* and *GATA3* expression. Finally, interleukin-2 (IL-2) signaling is known to drive the formation of Th1 precursor cells and also sustain Th1 responses during later stages of infection (36). Enhanced IL-2R signaling is also a key factor in programming CD8 T cells to become long lasting, functional memory cells and upregulation of IL-2 receptor beta (*IL2RB*) in vaccinated animals at 36 DPV suggests that the subunit vaccine may provide a cell-mediated immune memory (37).

IL-27 can stimulate Th1 cell differentiation and is linked to the activation of CD8 T cells (38). An alternative function of IL-27 includes modulating inflammatory responses during infection (39). The inhibition of *IL27RA* results in increased macrophage damage and inflammation during *Mycobacterium tuberculosis* infection (40). Caseonecrosis is frequently observed in the lungs of bison and cattle during *M. bovis* infection. The multifocal lesions present in the lungs have noted infiltration of macrophages and neutrophils often surrounding a core comprised of necrotic material, demonstrating excessive myeloid cell infiltration is involved in lung tissue damage (13, 41). The upregulation of *IL27RA* in vaccinated animals at 36 DPV in the present study may be related to reductions in inflammation and antibacterial activity of macrophages against *M. bovis*. Invasion of bovine cells by *M. bovis* has been demonstrated *in vitro*, suggesting the elimination of intracellular *M. bovis in vivo* is necessary for the clearance of infection (42, 43). An effective Th1-type immune response would be beneficial in eliminating infected cells and enhancing *M. bovis* clearance while limiting immunopathology. Together, these findings suggest that the adjuvanted protein subunit vaccine promoted a balanced, effective T-cell response based on the upregulation of interleukin receptors and key transcription factors.

The regulation of adhesion molecules involved in antigen presentation and co-stimulation, such as MHC Class I and *SPN*, has been associated with immune regulation and both were upregulated in vaccinated animals at 36 DPV. *SPN* (also known as *CD43*) promotes T cell activation and proliferation and has a role in neutrophil migration. *SPN* also has a protective role during *Staphylococcus aureus* infection, where monoclonal antibodies designed to target *SPN* impair phagocytosis, leading to increased bacterial burden, higher morbidity, and mortality, demonstrating that *SPN* has a role in effective immune responses by potentially enhancing phagocytic function (44). The MHC class I molecules are expressed in all nucleated cells and present endogenous antigens to CD8 cells, in which they bind and display viral peptides on their surface. The MHC class I molecule can then be recognized by cytotoxic T cells in order to destroy cells infected with viruses (45, 46). A chaperone protein, *TAPBP,* is involved in endogenous antigen presentation by MHC class I and functions crucially in peptide loading onto MHC class I molecules (47). The upregulation of *TAPBP* in vaccinated animals may underlie increased immune responses by CD8 T cells.

An ortholog of NK-lysin (LOC105004569) was a DET that was upregulated in vaccinated animals at 21 and 36 DPV. NK-lysins are multifunctional peptides found in cytolytic granules of cytotoxic T-lymphocytes and natural killer cells with antibacterial and antiviral activities (28, 48, 49). Earlier studies have demonstrated the antimicrobial action of bovine NK-lysin against pathogens associated with bovine respiratory disease, including *M. bovis*, *Histophilis somni*, *Pasteurella multocida*, and *M. haemolytica* (50–52). Our previous work has shown the ability of bovine NK-lysins to breach the plasma membrane permeability barrier of *M. bovis*, causing membrane depolarization and structural damage to the plasma membrane (50).

Co-expression analysis revealed a negative correlation between vaccination and the expression of transcripts involved in cytokine-mediated signaling and chemokine signaling pathways at 40 and 47 DPV. This suggests that BHV-1 may have an immunosuppressive effect at 40 DPV. *VIM* was one of the co-expressed transcripts with the highest treatment significance and module membership, where decreased expression of vimentin can inhibit NLRP3 inflammasome signaling and cytokine production (53). Transcripts in Module V, including *HCK* and *FGR*, were also negatively correlated to vaccination status. Synergistic regulation by these Src family kinases has been previously described in mice, where knockout of *HCK* and *FGR* in addition to *Lyn* results in decreased inflammatory effects due to inhibited cytokine production (54). Given that cytokine and chemokine signaling shape the inflammatory response, altered expression of these transcripts may influence immune cell activation and the inflammatory environment in vaccinated animals. Potentially, this leads to increased pathogen control while decreasing inflammation and immunopathology following *M. bovis* challenge.

The gene *LGALS3* was the most significantly dysregulated gene in spleen and was downregulated in vaccinated animals. *LGALS3* encodes Galectin-3, which has been described as a facilitator of viral attachment and entry as well as a stimulator of proinflammatory cytokines and chemokines to illicit an antiviral response (55–57). The ability of Galectin-3 to promote migration of immune cells to infection sites can exacerbate inflammation and tissue damage. Both small molecule inhibitors and monoclonal antibodies targeting Galectin-3 have potential for antiviral therapy as inhibition by a Galectin-3 antagonist resulted in reduced viral load in COVID-19 patients (56, 58). Galectin-3 knockout mice displayed reduced *Brucella abortus* loads as well as increased numbers of macrophages and neutrophils in spleen (59, 60). Galectin-3 also facilitates the delivery of antimicrobial *GBP-1* to pathogen containing vacuoles, which are intracellular compartments for microbial growth (61). GBPs are induced by proinflammatory stimuli, mainly interferons. In some cases, *M. bovis* infection can cause moderate splenitis, and the decreased expression of Galectin-3 and *GBP-1* in vaccinated animals could perhaps be associated with a reduction in inflammation of the spleen (5). Given that the spleen has a range of immunological functions (e.g., filtering pathogens from blood, antigen trapping and regulation of T and B cell response), vaccination may have had an influence on splenic function and aided in recovery from *M. bovis* infection (62).

The present study utilizes transcriptomics to characterize immune response to vaccination and the mechanisms contributing to reduced clinical disease. Robust transcriptional regulation of immune-related pathways was primarily observed at 36 DPV with significant upregulation of cell adhesion, Th1/Th2/Th17 cell differentiation, and antigen processing and presentation. The upregulation of T helper differentiation pathways suggests a maturing immune response, while enhanced antigen presentation and cell adhesion may describe increased immune cell trafficking and interactions due to vaccination. Trafficking of immune cells potentially leads to the recruitment of immune cells to interact at sites where they are needed to fight infection. Spleen-specific regulation included transcripts involved in innate immune response and inflammation, such as *LGALS3* and *GBP-1*. It is important to note that a small sample size (*n* = 4–5) was used in this study and future work should consider including larger sample sizes to account for individual variability in vaccine response. In addition, BHV-1 was used in the study to replicate a co-infection model, which may have introduced off-target effects that confound our interpretation of the immune response to *M. bovis* inoculation. These findings are the first to describe the transcriptomic response to *M. bovis* infection in bison and suggest that the protein subunit elicited a broad, temporally regulated immune response detected in both blood and tissue.

## Data Availability

The original contributions presented in the study are publicly available. The sequence files can be found in the NCBI repository under BioProject accession number PRJNA1274494 (https://www.ncbi.nlm.nih.gov/bioproject/?term=%20PRJNA1274494).
